# Dr. James M. O’Neill

**Published:** 1908-08

**Authors:** 


Dr. James M. O'Neill, of Buffalo, died at his home 381 Swan Street, July 15, 1908, aged 56 years. He was a native of Dublin, Ireland, and a graduate of Dublin University. He practised his profession in England for some years, but came to Buffalo about twenty-three years ago settling near the old Hydraulics, where he obtained prominence as a conscientious and skilful physician. He is survived by three daughters and four sons. Mrs. O'Neill died about three years ago.

